# Applying the Net-Benefit Framework for Analyzing and Presenting Cost-Effectiveness Analysis of a Maternal and Newborn Health Intervention

**DOI:** 10.1371/journal.pone.0040995

**Published:** 2012-07-19

**Authors:** Sennen Hounton, David Newlands

**Affiliations:** 1 Centre MURAZ, Bobo-Dioulasso, Burkina Faso; 2 Immpact, University of Aberdeen, Scotland, United Kingdom; National Institute for Public Health and the Environment, The Netherlands

## Abstract

**Background:**

Coverage of maternal and newborn health (MNH) interventions is often influenced by important determinants and decision makers are often concerned with equity issues. The net-benefit framework developed and applied alongside clinical trials and in pharmacoeconomics offers the potential for exploring how cost-effectiveness of MNH interventions varies at the margin by important covariates as well as for handling uncertainties around the ICER estimate.

**Aim:**

We applied the net-benefit framework to analyze cost-effectiveness of the Skilled Care Initiative and assessed relative advantages over a standard computation of incremental cost effectiveness ratios.

**Methods:**

Household and facility surveys were carried out from January to July 2006 in Ouargaye district (where the Skilled Care Initiative was implemented) and Diapaga (comparison site) district in Burkina Faso. Pregnancy-related and perinatal mortality were retrospectively assessed and data were collected on place of delivery, education, asset ownership, place, and distance to health facilities, costs borne by households for institutional delivery, and cost of standard provision of maternal care. Descriptive and regression analyses were performed.

**Results:**

There was a 30% increase in institutional births in the intervention district compared to 10% increase in comparison district, and a significant reduction of perinatal mortality rates (OR 0.75, CI 0.70−0.80) in intervention district. The incremental cost for achieving one additional institutional delivery in Ouargaye district compared to Diapaga district was estimated to be 170 international dollars and varied significantly by covariates. However, the joint probability distribution (net-benefit framework) of the effectiveness measure (institutional delivery), the cost data and covariates indicated distance to health facilities as the single most important determinant of the cost-effectiveness analysis with implications for policy making.

**Conclusion:**

The net-benefit framework, the application of which requires household-level effects and cost data, has proven more insightful (than traditional ICER) in presenting and interpreting cost-effectiveness results of the Skilled Care Initiative.

## Introduction

**Figure 1:**
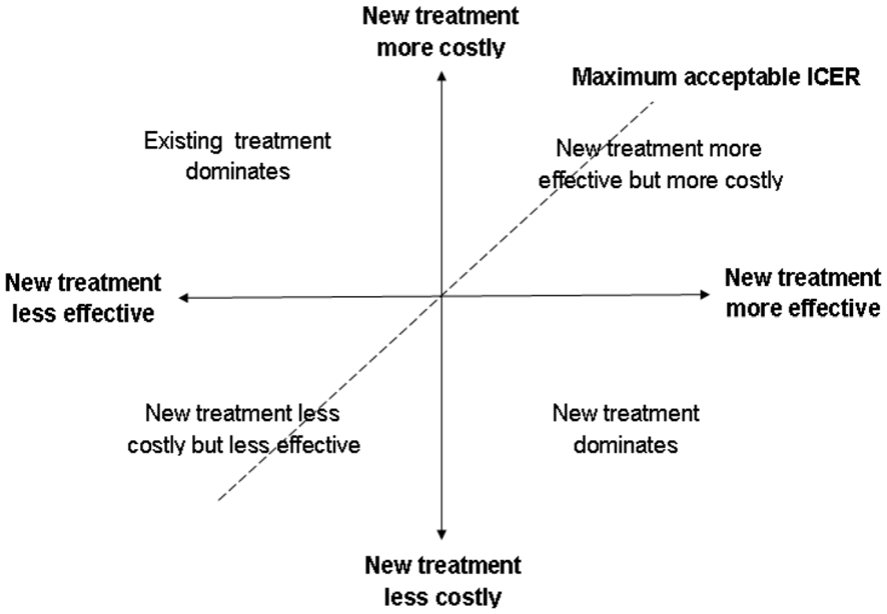
Cost-effectiveness plane.

Given the importance of determinants affecting access and uptake of maternal and newborn health [1], any analysis of effectiveness [2], [3] and cost-effectiveness needs to account for the known and unknown covariates. Economic evaluation in general, and cost-effectiveness analysis in particular, is an important element of evidence-based policy making in balancing health gains against the costs of interventions [4]. Cost effectiveness analysis in maternal and newborn health mostly revolves around the use of the standard Incremental Cost Effectiveness Ratio (ICER), which indicates the additional amount of money needed to obtain an extra unit of health gain or to prevent an adverse event compared to alternatives [5]–[6]. In few situations is there a clearly dominant intervention (the existing intervention is less effective and more costly or a new intervention is more effective and less costly). However, in most maternal and newborn health interventions (complex interventions often building on existing packages of activities and services), new interventions are often more effective and more costly and thus there is a trade-off of relative advantage or relative cost saving (Figure 1). In these circumstances, there is a need to estimate the maximum a provider (society or the health system) is willing to pay for an additional unit of health gain (averting one extra maternal or newborn death, or achieving one extra institutional delivery, etc.). When using the ICER, it is notoriously difficult to reliably build confidence intervals around the ICER estimates for inferential analysis [7], [8], and the maximum a provider is willing to pay is often unknown to analysts unless extensive willingness to pay surveys are regularly carried out [10]. We applied the net-benefit framework [11], [12], [13] to an observational maternal and newborn health study to assess the feasibility (including data requirements) and relative advantages in presenting and interpreting results of cost-effectiveness analysis. By collecting household costs and by transforming the dataset to have concurrent household-level effect and cost data, we could assess the importance of significant determinants on how the cost-effectiveness varies at the margin which is important for policy making and scaling up but which is not feasible with the standard ICER statistic.

## Methods

### Context and the Interventions

The data used for this work were derived from the evaluation of the Skilled Care Initiative in Burkina Faso, an observational study. The detailed information about the evaluation methods, the context and components of interventions in Ouargaye district (where the Skilled Care Initiative was developed and implemented), and Diapaga district (comparison area) can be found elsewhere [12], [13]. In summary, Ouargaye and Diapaga are two remote and rural districts in East and Centre East of Burkina Faso in West Africa. As part of the evaluation of the Skilled Care Initiative, an extensive mapping and description of interventions in both districts during the lifetime of the Skilled Care Initiative (2001–2005) was conducted. The most distinctive difference between the mix of interventions in the two districts was the comprehensive community mobilization developed in Ouargaye district and it is on that basis that we estimated and reported the additional cost per extra institutional delivery between the districts and the maternal and perinatal mortality advantage in the intervention district [13], [14]. The unit of analysis was a health district: Ouargaye district (nearly 250,000 inhabitants, where the Skilled Care Initiative project was implemented) is the ‘*intervention*’ and Diapaga district (nearly 350,000 inhabitants with the standard package of activities in Burkina Faso health districts) is the comparator or ‘*status quo’*.

### Data Collection and Analysis

Data were collected from January to July 2006. A geo-referenced census was conducted in the intervention district (Ouargaye) and a comparison district (Diapaga). Information were collected from all women aged 12–49 with experience of pregnancy in the survey referenced period (2001–2005), on the number of pregnancies, pregnancies lasting more than six months, place of delivery, delivery attendant and newborn survival during the five years prior to the interview.

#### Computing the traditional incremental cost effectiveness ratio (ICER)

In addition we costed the standard provision of maternal care in both districts to analyze the main cost structure in both districts, and estimated the incremental cost per delivery based on the assumption that the 30% increase in institutional delivery recorded in Ouargaye district compared to a 10% increase in Diapaga district was mainly attributable to the comprehensive community mobilization of the Skilled Care Initiative [13], [14].

#### Constructing dataset for net benefit regression

Data were collected from all women of reproductive age with experience of delivery in the 6 weeks prior to the survey, on costs borne by women and families for institutional delivery, as well as household revenues. A database was subsequently constructed using the household cost survey with geo-coordinates, asset ownership quintiles, education of head of household, distance to health facility, household revenues, perinatal death, and family size. The health system in Burkina Faso is very centralized and we assumed the standard maternal care in both remote and rural districts was the same, although differences have been documented among health centers across both districts [14]. We estimated for each household with an institutional delivery in the intervention district (Ouargaye) the household cost of an institutional delivery and the estimated incremental cost to the health system of an institutional delivery to derive a cost for institutional delivery from a societal perspective. For households with no institutional delivery, only the estimated incremental cost to the health system of an institutional delivery was attributed to the households. Conversely, in the comparison district (Diapaga), we attributed to each household with an institutional delivery the household cost of an institutional delivery but no incremental cost from the health system perspective. This does not mean there was no cost from the health system perspective in Diapaga but that the average standard cost from the health system will cancel out between the intervention and comparison sites.

Assuming we have household-level effect and cost data, the traditional equation ΔC/ΔE (ICER) where ΔC is the incremental cost and ΔE the incremental effect can be re-arranged by multiplying each arm of the equation by ΔE. The result is ΔC  =  ΔE * ICER and for any ceiling ratio Ro, ΔC  =  ΔE * Ro. Thus, a net-benefit statistic can be computed as follows: ΔE*Ro − ΔC  =  ΔNB. We thus constructed for each observation (household) of the household cost survey an individual net-benefit statistic. The expression of an individual (or household) net-benefit NBi  =  ΔEi*Ro − ΔCi is similar to a traditional linear regression equation Y  =  α + δX_i_ + ε_i_ where Y is the dependent variable, α is the intercept, δ the coefficient on an explanatory variable X (continuous variable or dummy variable taking the value 1 for a positive outcome and 0 for a negative outcome for example) and ε_i_ the standard error.

For the evaluation of the Skilled Care Initiative, the household net-benefit was modeled as NB_i_  =  α+δSCI_i_ + ε_i_ where NB_i_ is the net-benefit for each household, α is the intercept, δ the coefficient (incremental net benefit ) on the intervention (SCI taking the value zero for households in the intervention district and 1 for households in the comparison district), and ε_i_ the standard error. These statistics are obtained by running an Ordinary Least Squares (OLS) regression. The interpretation is straightforward. When the difference is greater than zero, it means that the incremental cost for one additional unit of effectiveness (in this case institutional delivery) is below the ceiling ratio Ro (the maximum the provider is willing to pay). Then the SCI will be deemed cost-effective in Ouargaye district in comparison to Diapaga district. Similarly, if the coefficient is negative, then the incremental cost for one additional unit of effectiveness is above the Ro and the standard health system in Diapaga district will be deemed more cost-effective than the SCI intervention.

The basic model, NB_i_  =  α+δSCI_i_ + ε_i_, can then be further improved to include important covariates and therefore allow the examination of the marginal impact of these covariates on incremental cost effectiveness. The final model may look like:

NB_i_  =  α + ∑^P^_j = 1_ β_j_ x_ij_ + δSCI_i_ + ε_i._where NB_i_ is the summation of the interaction between the treatment dummy (SCI, coded yes or no) and the covariates, α is the intercept, β_j,_ the parameter which indicates the average change in NB_i_ that is associated with a unit change in X (covariate) whilst controlling for the other explanatory variables, δ the coefficient on the intervention (SCI), and ε_i_ the standard error.

#### Assessing uncertainties: cost-effectiveness acceptability curves

**Figure 2:**
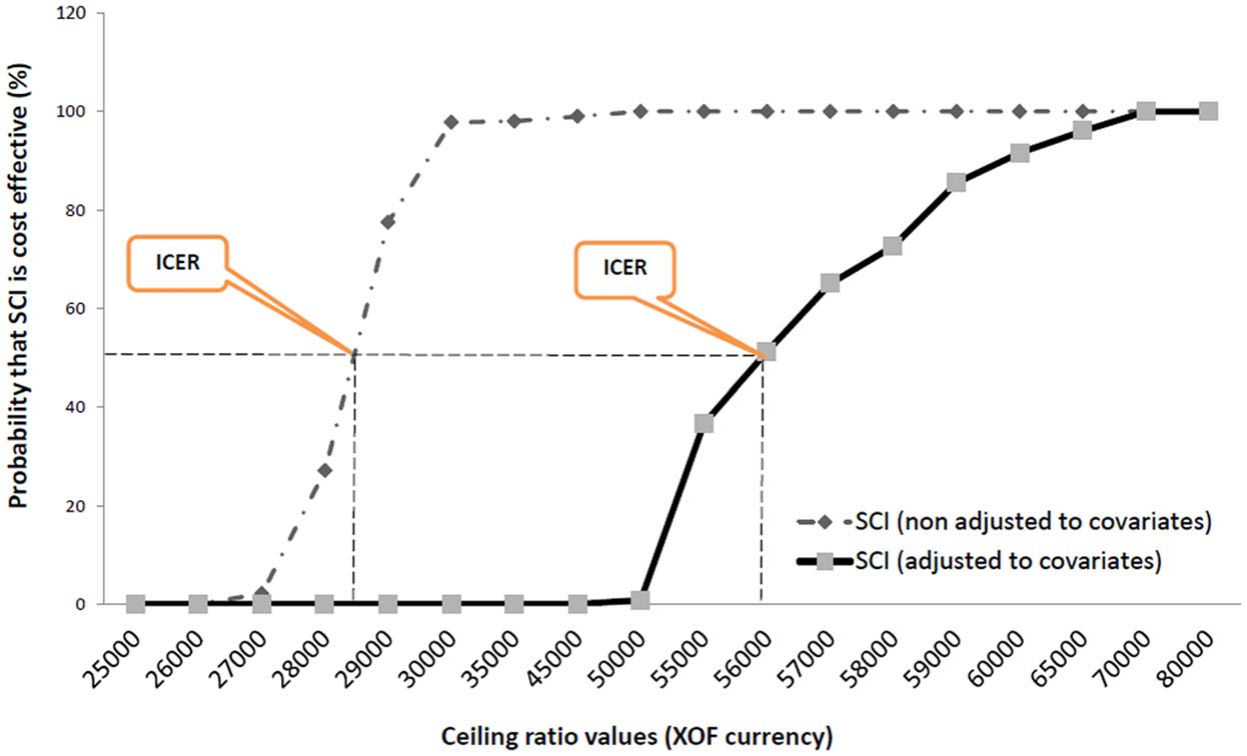
Cost-effectiveness acceptability curves (CEAC), Skilled Care Initiative (non-adjusted to covariates).

A cost-effectiveness acceptability curve graphically represents the levels of certainty around the cost-effectiveness ratio of two interventions by plotting hypothetical estimates of ceiling ratios to the probability that an intervention is cost-effective [15], [16], [17], [18]. The p-values obtained from the net benefit regression are two-sided but only one-sided values are needed to test whether the incremental net-benefit is positive (the new intervention is preferred) or negative (the standard intervention is preferred) so the regression p-values were divided by two. For negative incremental net-benefits the probability that the new intervention is preferred equals the one-sided p-value, and for positive incremental net-benefits, the probability that the new intervention is preferred equals 1 minus the one sided p-value. The cost-effectiveness acceptability curves are obtained by plotting a graphical representation of hypothetical ceiling ratios against the probability that the intervention is cost-effective and can be used to construct confidence intervals around the ICER (Figure 2).

### Ethical Considerations

The study was approved by the Burkina Faso Health Sciences Ethical Review Board. Informed consent from all participants (head of household or adult member of the household) involved in our study was obtained verbally prior to the interviews. Written consent was not deemed necessary by the ethics committees as the survey is a classic Demographic and Health Survey (DHS) type survey and data were to be analyzed anonymously and no individual data analysis was proposed. The investigation team relied on trained supervisors to ensure consent was secured and the ethics committees approved this consent procedure.

## Results

### Cost-effectiveness Analysis using the Standard ICER

Table 1 presents the sample statistics of the cost-effectiveness analysis of the Skilled Care Initiative. The ICER value represents the additional resources required per additional delivery in a health facility. Summary estimates are presented using international dollars. International dollars are adjusted for differential purchasing power across countries. In Burkina Faso at the time of the intervention one international dollar was equivalent to 167 CFA (West African Francs). As we can observe, the ICER estimate varies significantly across subsets of the population by covariates. For example, the incremental cost for one extra institutional delivery among people with no education was I$168 compared to I$84 among people with some level of education (at least primary school level). The incremental cost of one extra institutional delivery among people within 5 km of the closest health facility was I$303 compared to I$383 among people at least 5 km away from the closest heath facility. These results confirm the existence of important subgroups, and thus the importance of assessing how these covariates affect the overall cost-effectiveness of the Skilled Care Initiative. This could only be achieved through a joint probability distribution with all covariates which is possible with the net-benefit approach but not with stratified analysis of the traditional ICER.

**Table 1:** Sample statistics from cost-effectiveness analysis of the SCI, Ouargaye and Diapaga districts, Burkina Faso.

Group variables	Mean	SD	SE
**Overall analysis**			
*Comparison district (N = 48 272)*			
Cost*	1042	–	–
Effect** (%)	31.5	0.464	0.002
*Intervention district (N = 40 469)*			
Cost*	4576	–	–
Effect** (%)	44	0.496	0.002
Cost increment	3534	–	–
Effect increment (%)	12.4		0.003
*Sample ICER* ***	**28430 (I$170)**		

* Average cost (heath system perspective) for maternal health provision in West Africa Francs.

** Institutional delivery.

*** (Ratio of incremental cost to incremental effect).

Summary estimates are presented in the manuscript using international dollars (international dollars are ones adjusted for differential purchaising power. In Burkina Faso at the time of the intervention it was around 167, WHO, 2005) to enable easy understanding of international readers.

### Applying the Net-benefit Framework: Linearization of the ICER

Table 2 presents the coefficients of the net-benefit estimates, obtained from a classic (OLS) regression procedure, with net-benefit as outcome and the intervention dummy (SCI) as independent variable. The coefficient for the intervention (in this case the Skilled Care Initiative) corresponds to the incremental net-benefit and is equivalent to the standard ICER.

**Table 2:** Simple net-benefit regression estimates with different ceiling ratios, Skilled Care Initiative, Burkina Faso.

Group variables	Mean	SD	SE			
**Overall analysis**						
Cost increment	3534	–	–			
Effect increment (%)	12.4		0.003			
Sample ICER***	**28430 (I$170)**					
**Stratified analysis by education**						
*None*						
Cost difference	3534					
Effect difference (%)	13	–	0.003			
Sample ICER	**28 115 (I$168)**					
*Some*						
Cost difference	3534					
Effect difference (%)	25	–	0.015			
Sample ICER	**13 950 (I$84)**					
**Stratified analysis by distance**						
*Less than 5 km*						
Cost difference	3534					
Effect difference (%)	7		0.004			
Sample ICER	**50 558 (I$303)**					
*More than 5 km*						
Cost difference	3534					
Effect difference (%)	5.5		0.005			
Sample ICER	**63 906 (I$383)**					
**Incremental net-benefit**	Net Monetary Benefit coefficients (SE)					
Ceiling ratios **Overall**	**Edu = None**	**Edu = Some**	**Dist:>5 km**		**Dist: ≤5 km**	
*Ro = 0–3534 (0.0)*	*−3534 (0.0)*	*−3534 (0.0)*	*−3534 (0.0)*		*−3534 (0.0)*	
*R = 15 000–1670 (48)*	265 (226)	*−*1648 (50)	*−*2705 (76)		*−*2485 (59)	
*R = 25 000–426 (80)*	2798 (377)	*−*391 (83)	*−*2152 (126)		*−*1786 (98)	
*R = 35 000 817 (113)*	5331 (528)	867 (116)	*−*1599 (176)		*−*1086 (137)	

Edu: Education, Dist: Distance to health facility.

Summary estimates are presented in the manuscript using international dollars (international dollars are ones adjusted for differential purchaising power. In Burkina Faso at the time of the intervention it was around 167) to enable easy understanding of international readers.

We used different ceiling ratios around the value of the computed ICER (approximately I$170) and as the ceiling ratios increase moving from zero to infinity, the coefficients (incremental net-benefits) obtained from the OLS regression on the intervention and covariates increase (Tables 3 and Table 4 in Appendix S1).

**Table 3:** Simple net-benefit regression estimates with different ceiling ratios, Skilled Care Initiative, Burkina Faso.

Explanatory Variables	NMB	NMB	NMB	NMB	NMB	NMB
	With Ro = 0^a^	With R 15000	With R 25000	With R 35000	With R 45000	With R 55000
	[SE]	[SE]	[SE]	[SE]	[SE]	[SE]
	(p-value)	(p-value)	(p-value)	(p-value)	(p-value)	(p-value)
						
**Constant term**	*−*1042	3682	6832	9981	13130	16280
	[0]	[33]	[55]	[76]	[98]	[120]
	(0.000)	(0.000)	(0.000)	(0.000)	(0.000)	(0.000)
						
**Intervention strategy (SCI)**	*−*3534	−1670	−426	816	2060	3203
	[0]	[48]	[80]	[113]	[145]	[178]
	(0.000)	(0.000)	(0.000)	(0.000)	(0.000)	(0.000)
						
**R^2^ (adjusted)**	1	0.013	0.000	0.001	0.002	0.004
**F (1, 88741)**	–	1185	27.8	52	200	345
**Prob > F**	–	<0.000	<0.000	<0.000	<0.000	<0.000
						

a When Ro = 0, NMB  =  −Cost.

Summary estimates are presented in the manuscript using international dollars (international dollars are ones adjusted for differential purchaising power. In Burkina Faso at the time of the intervention it was around 167) to enable easy understanding of international readers.

**Figure 3:**
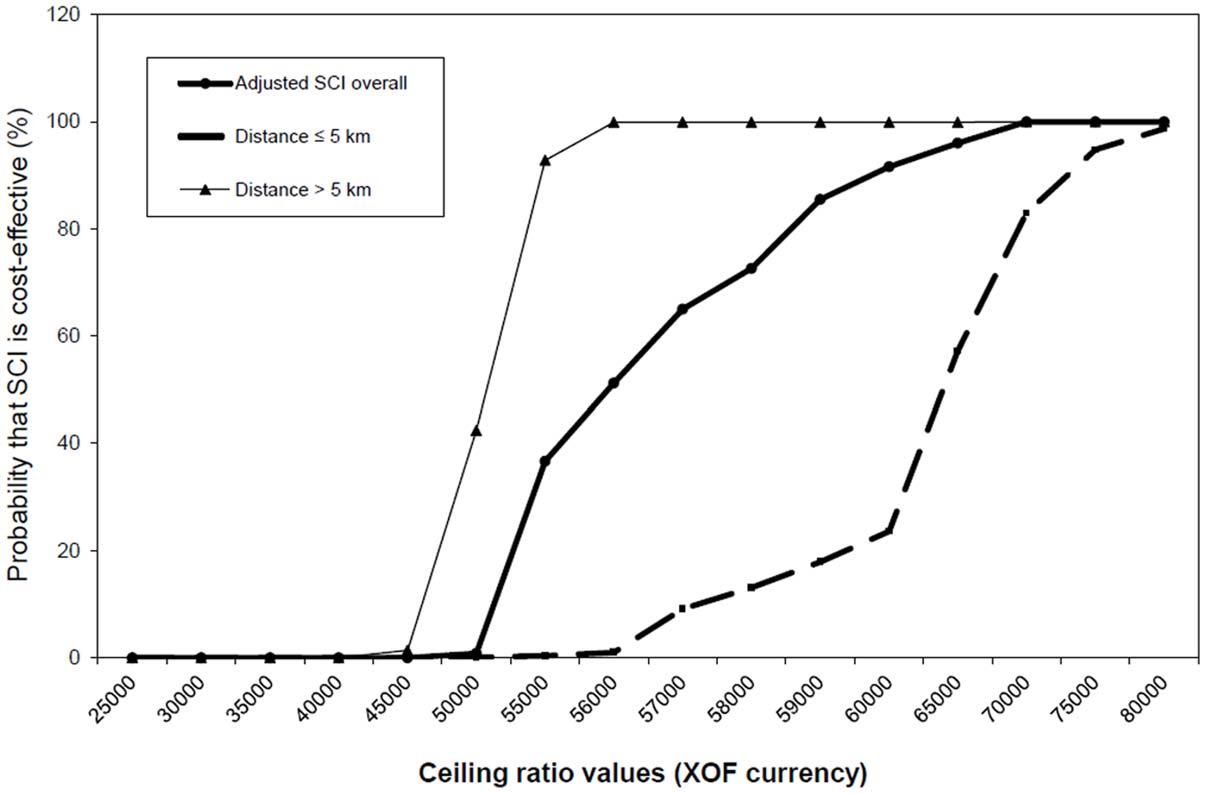
Non adjusted cost-effectiveness acceptability curves, Skilled Care Initiative overall and with covariate distance.

Tables 3 and 4 in Appendix S1 illustrate how the coefficient changes from a negative value (negative incremental net-benefit, the intervention not reaching an acceptable level of cost-effectiveness) to a positive value (positive incremental net-benefit, the intervention deemed cost-effective). In Table 3, for example, the probability that the intervention is cost-effective varies from zero to 98% in a narrow interval between the values I$150 and I$180 (international dollars). Figure 2 highlights one of the major advantages of the net-benefit framework, that we can see clearly how different the ICER values are when adjusted or not to the covariates. It is therefore important to adjust the ICER to important subgroups. One can observe in Table 5 in Appendix S1 and Figure 3 that the probability of the intervention being cost-effective when the ceiling ratio is 60,000 CFA (approximately I$360 international dollars) is only 24% for households living 5****km or less from a health facility, whilst the corresponding probability for households living 5 km or more from a health facility is nearly 99%. This is a major difference to the standard ICER approach where it is not possible to indicate the probability that the intervention is cost-effective adjusting for other covariates.

Putting the results in Figure 3 into practice, one can say that if policy makers in rural Burkina Faso were prepared to invest I$360 to achieve an extra institutional delivery the results will vary significantly for households whether they are situated closer (less than 5 km) or further away (more than 5 km) from a health facility. This is very important information for the scaling up process.

### Assessing the Effects of Covariates on Marginal Cost-effectiveness

Given that education, distance to the closest health facility and asset ownership are major determinants of the effects of the Skilled Care Initiative, it is critical to be able to assess the effects on the marginal cost-effectiveness of adjusting for these important covariates.

When running the OLS regression with net-benefit as the dependent variable, and education, distance to closest health facility, and asset quintiles as independent variables, the variability of the cost-effectiveness acceptability curves (CEAC) by covariates (Figures 2 and 3) highlights the importance of adjusting the cost-effectiveness analysis by covariates. We can explore for each covariate the difference in the probability that the Skilled Care Initiative (SCI) is cost-effective when adjusted or not for the selected covariate but only a joint probability distribution with all covariates is appropriate. As for any regression analysis, we can assess the variability accounted for by the covariates and their probability of correlation with the intervention. It is important to point out that although education, distance to health facilities, and asset ownership are well documented determinants of health outcomes, these three factors appear to explain only a small fraction of the cost-effectiveness of the Skilled Care Initiative in rural Burkina Faso (adjusted R^2^ around 13%).

Table 6 in Appendix S1 presents the results of the regression analysis with interaction terms. When running an OLS regression using the net-benefit framework with the interaction terms, the main interest is in the coefficients of the intervention dummy (SCI) which corresponds to the incremental net-benefit. The magnitude and significance of the coefficients on the interaction terms (interaction between the covariates and the intervention dummy) indicate how cost-effectiveness of the Skilled Care Initiative is expected to vary at the margin. A large and statistically significant coefficient on an interaction will usually point to an important population subgroup. From Table 6 in Appendix S1, we may infer that there is an interaction between the intervention and the education dummy (first interaction term) and between the intervention dummy and asset ownership dummy (third interaction term) but no interaction between the intervention dummy and distance to health facility (second interaction term). However, the observed interactions may be due to collinearity between covariates and from this point we may decide to include or omit a covariate based on prior knowledge or practical implication, based on model diagnosis. The largest and most significant coefficients for collinearity are observed between distance and asset ownership, and between the intervention dummy and asset ownership quintiles (data not shown). We conclude that distance was the most important covariate among the three analyzed.

## Discussion

By applying the net-benefit framework, we were able to show that the probability (adjusting for the covariates) of the Skilled Care Intervention to achieve one extra institutional delivery in rural Burkina Faso when the ceiling ratio is approximately I$360 is only 24% for households living 5****km or less from a health facility, whilst the corresponding probability for households living 5 km or more from a health facility is nearly 99%. This major piece of information (variability in probability in cost-effectiveness by important sub-groups) together with the possibility of providing some level of certainty (confidence intervals) around the ICER estimate would not have been possible just by using the traditional ICER method for this observational study. As pointed out by Hosh JS et al [2] the existence of important sub-groups (which is the case for maternal and newborn health) affects how the cost-effectiveness varies at the margin and need to be accounted for when analyzing and interpreting cost-effectiveness results.

In this study, the small degree of variability explained by the three determinants (household education level, distance, asset ownership) may be explained by the existence of a more important unknown variable and/or the content validity of our constructed metrics of the covariates. It is well documented that the mix of assets affects the constructed wealth index and the constructed index may not reflect consumption and socio-economic status well. Furthermore, the remoteness of the study context and similarity in household characteristics across the districts may result in a very low variability of the asset ownership quintiles [19].

Our results point first to the importance of covariates (Figure 2). There is a clear difference between the probability that the intervention is cost-effective at a given ceiling ratio when adjusted or not adjusted to the covariates. Distance appears to be the most important covariate in our study context. Cost effectiveness of the Skilled Care Initiative varies significantly by the average distance of households to the nearest health facility (after adjusting for all covariates).

Unlike some developed countries where extensive studies have been conducted to generate appropriate ceiling ratios for health outcomes and where there are evaluation bodies, such as NICE in the United Kingdom and CADTH in Canada, that continually update these estimates, researchers and policy makers in the developing world have almost no information about their contextual ceiling ratios. In these settings, the identification of an ICER point estimate where there is no information on uncertainties may be of little use as policy makers will have no basis to judge whether or not the ICER is good value for money. It is thus important to revise the current practice of the presentation and interpretation of cost-effectiveness analysis results of maternal and newborn interventions to improve evidence-based decision making.

There are increasing opportunities through household surveys (Demographic and Health Surveys, Multiple Indicators Cluster Surveys, Living Standard Measurement Surveys) to construct person-level or user-level (household, individual) effect and cost (willingness to pay, out-of-pocket expenditures) data, necessary for application of the net-benefit framework.

A cost-effectiveness analysis requires the comparison of like with like, which is not often the case with observational studies. However, in spite of inherent differences between any two populations, comparability of settings on specific characteristics within or between subsets of populations is important before the implementation of an intervention can be evaluated [20]. The analyst will need to identify and adjust for significant covariates or important subgroups within a population and descriptive statistics such mortality ratios and ICER estimates only do not account for these important covariates [3]. Lastly, constructing the database from an observational study to apply the net benefit framework requires careful planning. Typically, a clinical trial database will have patient-level effect and cost data. In observational studies, only effect data are usually collected and additional (often separate) costing exercise conducted, sometimes through modeling, sometimes with field data collection. We were able to demonstrate that by carefully designing an observational study to collect concurrent patient-level (household or individual) effect and cost data we can apply the net-benefit framework in maternal and newborn health.

### Conclusions

We have demonstrated that the net-benefit framework is applicable to observational studies common in maternal and newborn health and that the cost effectiveness of a maternal and newborn health intervention varies by important covariates (sub groups). By adjusting the intervention cost-effectiveness results to the covariates, we were able to identify distance to health facilities as an important determinant of the cost-effectiveness analysis. We could not have reached the same conclusion employing the traditional ICER approach. These advantages in the presentation and interpretation of cost-effectiveness analysis results, and especially in providing information on the marginal cost-effectiveness of important covariates, necessitate that we revise the traditional methods and tools of maternal and newborn health household surveys to include household out-of-pocket expenditure for health outcomes. The recent development of household cost surveys alongside Demographic and Health Surveys (such as out-of-pocket expenditure data from National Health Accounts) offers broader opportunities for the construction of concurrent household level effect and cost datasets and application of the net-benefit framework for assessing the cost-effectiveness of maternal and newborn health interventions.

## Supporting Information

Appendix S1 Contains Table 4 (Using the net-benefit regression results to create cost-effectiveness acceptability curves), Table 5 (Cost-effectiveness acceptability curves from the net-benefit regression results with distance as covariate) and Table 6 (Covariates adjusted net-benefit regression estimates with different ceiling ratios, interactions).
(DOCX)
