# Supplementary material for: Applying the Net-Benefit Framework for Analyzing and Presenting Cost-Effectiveness Analysis of a Maternal and Newborn Health Intervention
Source: PLoS One. 2012 Jul 19;7(7):e40995. doi: 10.1371/journal.pone.0040995 (PMC3400570; doi:10.1371/journal.pone.0040995)
Supplement: Appendix S1 — Contains Table 4 (Using the net-benefit regression results to create cost-effectiveness acceptability curves), Table 5 (Cost-effectiveness acceptability curves from the net-benefit regression results with distance as covariate) and Table 6 (Covariates adjusted net-benefit regression estimates with different ceiling ratios, interactions). (DOCX) [file pone.0040995.s001.docx]

**Appendix S1. Tables 4, 5 and 6**

Table 4 Using the net-benefit regression results to create cost-effectiveness acceptability curves Skilled Care Initiative, Burkina Faso

| Values of  ceiling ratio | Treatment (intervention) coefficients | | One sided p-value | Probability of cost-effectiveness  (OLS regression) |
| --- | --- | --- | --- | --- |
|  | Estimates | P-values |  | % |
| 0 | - 3534 | 0.000 | 0.000 | 0 |
| 15000 (I$90) | - 1670 | 0.000 | 0.000 | 0 |
| 20000 (I$120) | - 1048 | 0.000 | 0.000 | 0 |
| 25000 (I$150) | - 426 | 0.000 | 0.000 | 0 |
| 26000 (I$156) | - 302 | 0.000 | 0.000 | 0 |
| 27000 (I$162) | - 177 | 0.042 | 0.021 | 2.1 |
| 28000 (I$168) | - 54 | 0.554 | 0.277 | 27.7 |
| 29000 (I$174) | 70 | 0.451 | 0.225 | 77.5 |
| 30000 (I$180) | 195 | 0.044 | 0.022 | 97.8 |
| 35000 (I$210) | 816 | 0.000 | 0.000 | 99.9 |
| 40000 (I$240) | 1438 | 0.000 | 0.000 | 99.99 |
| 45000 (I$269) | 2060 | 0.000 | 0.000 | 99.99 |
| 50000 (I$300) | 3302 | 0.000 | 0.000 | 99.99 |

I$ (international dollars)

Table 5 Cost-effectiveness acceptability curves from the net-benefit regression results of the Skilled Care Iinitiative (Distance covariate)

Distance ≤ 5 km Distance > 5 km

| Values of  ceiling ratio | Treatment (intervention) coefficients | | One sided  p-value | Probability of cost-effectiveness | Treatment (intervention) coefficients | | One sided  p-value | Probability of cost-effectiveness |
| --- | --- | --- | --- | --- | --- | --- | --- | --- |
|  | Estimates | P-values |  | % | Estimates | P-values |  | % |
| 0 | - 3534 | 0 | 0 | 0 | - 3534 | 0 | 0 | 0 |
| 40000 (I$240) | - 1323 | 0 | 0 | 0 | - 736 | 0 | 0 | 0 |
| 45000 (I$270) | - 1046 | 0 | 0 | 0 | - 387 | 0.028 | 0.014 | 1.4 |
| 50000 (I$300) | - 770 | 0.002 | 0.001 | 0.1 | - 37 | 0.849 | 0.424 | 42.4 |
| 55000 (I$330) | - 493 | 0.075 | 0.0037 | 0.4 | 313 | 0.145 | 0.072 | 92.8 |
| 60000 (I$360) | - 217 | 0.473 | 0.236 | 23.6 | 662 | 0.005 | 0.002 | 99.8 |
| 65000 (I$390) | 59 | 0.856 | 0.428 | 57.2 | 1012 | 0.000 | 0.000 | 99.9 |
| 70000 (I$420) | 336 | 0.340 | 0.170 | 83 | 1361 | 0.000 | 0.000 | 99.99 |
| 75000 (I$450) | 612 | 0.105 | 0.052 | 94.8 | 1711 | 0.000 | 0.000 | 99.99 |
| 80000 (I$480) | 888 | 0.027 | 0.013 | 98.7 | 2061 | 0.000 | 0.000 | 99.99 |

I$ (international dollars)

Table 6 Covariates adjusted net-benefit regression estimates with different ceiling ratios, interactions, Skilled Care Initiative, Burkina Faso

| N=  Explanatory  Variables | NMB  With Ro=0^a^  [SE]  (p-value) | NMB  With R 15000  [SE]  (p-value) | NMB  With R 25000  [SE]  (p-value) | NMB  With R 35000  [SE]  (p-value) | NMB  With R 45000  [SE]  (p-value) |
| --- | --- | --- | --- | --- | --- |
| Constant term | - 1042  [0] (0.000) | 3940  [83] (0.000) | 7261  [137] (0.000) | 10583  [193] (0.000) | 13904  [248] (0.000) |
| **Covariates**  Education | - 3087  [2266] (0.173) | 1379  [118] (0.000) | 2299  [196] (0.000) | 3218  [275] (0.000) | 4138  [353] (0.000) |
| Distance | 1.4 E-10  [0] (1.000) | - 4289  [65] (0.000) | - 7148  [108] (0.000) | - 10007  [152] (0.000) | - 12866  [195] (0.000) |
| Assets ownership | 1.4 E-11  [0] (1.000) | 1038  [24] (0.000) | 1730  [41] (0.000) | 2422  [58] (0.000) | 3115  [75] (0.000) |
| **Interactions**  Interaction  SCI *education | 3.8 E-11  [0] (1.000) | 1813  [25] (0.000) | 3022  [359] (0.000) | 4230  [502] (0.000) | 5439  [645] (0.000) |
| Interaction  SCI *distance | - 2.2 E-10  [0] (1.000) | - 64  [94] (0.494) | - 107  [157] (0.494) | - 150  [220] (0.494) | - 193  [283] (0.494) |
| Interaction  SCI *Assets | -1.0 E-10  [0] (1.000) | - 683  [38] (0.000) | - 1138  [63] (0.000) | - 1593  [88] (0.000) | - 2048  [114] (0.000) |
| **Intervention strategy (SCI)**  R^2^ (adjusted)  F (1, 88548)  Prob > F | - 3534  [0] (0.000)  1.000  4 E+17  < 0.000 | -1447  [148] (0.000)  0.138  2027  < 0.000 | - 56  [246] (0.819)  0.127  1837  < 0.000 | 1335  [344] (0.000)  0.127  1841  < 0.000 | 2726  [443] (0.000)  0.129  1865  < 0.000 |
